# *Zizania latifolia* Cell Wall Polysaccharide Metabolism and Changes of Related Enzyme Activities during Postharvest Storage

**DOI:** 10.3390/foods11030392

**Published:** 2022-01-29

**Authors:** Jing Huang, Weijie Wu, Xiangjun Fang, Hangjun Chen, Yanchao Han, Ben Niu, Haiyan Gao

**Affiliations:** 1 Key Laboratory of Post-Harvest Handling of Fruits, Key Laboratory of Fruits and Vegetables Postharvest and Processing Technology Research of Zhejiang Province, Zhejiang Academy of Agricultural Sciences, Food Science Institute, Ministry of Agriculture and Rural Affairs, Hangzhou 310021, China; jhuangdcm@163.com (J.H.); wuweijie87@163.com (W.W.); fangxiangjun2004@163.com (X.F.); hanhanzhixing@126.com (Y.H.); newben1989@163.com (B.N.); 2 Key Laboratory of Postharvest Preservation and Processing of Fruits and Vegetables, China National Light Industry, Hangzhou 310021, China

**Keywords:** *Zizania latifolia*, cell wall polysaccharide, cell wall metabolic enzymes, Pearson’s correlation

## Abstract

The metabolism of polysaccharides in the *Zizania latifolia* cell wall helps maintain the postharvest quality during storage. Fresh *Z. latifolia* was stored at 4 °C and 25 °C to evaluate the hardness, cell wall polysaccharide composition, cell wall structure, active ingredients, and cell wall metabolism-related enzyme activities. The results showed that hardness declined concomitantly with an increase in water-soluble pectin content during storage, as well as with a decrease in propectin and cellulose contents. Correlation analysis showed that lower activities of cell wall-degrading enzymes, such as polygalacturonase, cellulase, and β-galactosidase in *Z. latifolia* stored at 4 °C, were associated with lighter fiberization and greater hardness, compared with those stored at 25 °C. Additionally, the results of infrared spectroscopy showed that texture softening may be attributed to a decrease in the degree of esterification of water-soluble polysaccharides at 25 °C compared to that at 4 °C.

## 1. Introduction

*Zizania latifolia* is native to Asia and belongs to the Gramineae family. *Z. latifolia* contains high amounts of various nutrients such as proteins, polyphenols, vitamins, and flavonoids [1]. It is a popular aquatic vegetable for consumption. As the main aquatic vegetable cultivated in China, *Z. latifolia* is appreciated by consumers for its delicate texture, refreshing taste, unique flavor, and nutritional value. It has various medicinal applications and health benefits [2]; however, *Z. latifolia* is highly perishable and susceptible to water loss, shrinkage, and lignification during storage [3]. *Z. latifolia* is highly prone to lignification after harvesting, which is attributed to the accumulation of lignin and polysaccharides in the cell wall.

Cell-wall polysaccharides, including pectin, hemicellulose, and cellulose, are important structural components and play a key role in maintaining the structure of fruit cells [4]. Studies of fruit textural properties focused on the enzymatic basis of cell wall modification, especially on enzymes involved in cell wall disassembly [5]. Previous research has explored the intrinsic relationship between textural properties and fruit cell wall polysaccharides by investigating the effects of cell wall modifying enzymes in different fruits, such as apricot [6], longan [7], kiwifruit [8], and loquat [9]. One of the causes of texture deterioration could be the metabolism of cell wall components [10], as the composition of the polysaccharides differs depending on the synergistic effect of multiple enzymes, including pectin methylesterase (PME), polygalacturonic acid (PG), and β-glucosidase (β-gal) [11]. However, only the lignification was evaluated in radiation preservation, while *Z. latifolia* was treated with modified atmosphere preservation [12]; there are limited studies represented in the literature about the cell wall polysaccharides.

*Z. latifolia* deterioration during storage is a complex process, usually accompanied by changing of water content and polysaccharide, which serve as the main contents supporting cell wall structure during the postharvest stage [8]. The purpose of this study was to explore the effect of cell wall metabolism on the dehulled *Z. latifolia* during postharvest storage at 4 °C and 25 °C by measuring the hardness, cell wall polysaccharide composition, and changes in corresponding enzyme activity. *Z. latifolia* was monitored at 4 °C and 25 °C to simulate the postharvest storage in the refrigerator and at room temperature, respectively.

## 2. Materials and Methods

### 2.1. Experimental Materials

*Z. latifolia* was harvested from Jiaxing, Zhejiang Province, China. All samples were of uniform maturity, color, and were not physically damaged. Two groups were stored at 4 °C and 25 °C, respectively. Three independent trials (12 samples per replicate) were carried out. Those stored at 4 °C were sampled five times (on days 0, 8, 16, 24, and 32). To account for the effects of microbial contamination, *Z. latifolia* stored at 25 °C was sampled three times on days 0, 8, and 16. All analyses were sampled once on 0 d. The collected samples were stored at −40 °C before analysis.

### 2.2. Hardness and Surface Hardness Measurements

Hardness was determined as described by Qi et al. [13]. Briefly, the direction of the probe was oriented perpendicular to the axis of the shoots from the equatorial region, where surface hardness (SH) was determined. A 1 cm thick disk was cut from the equatorial region of *Z. latifolia*. With the flat side of the disk horizontal, a 3 cm × 1 cm × 1 cm thick rectangular prism was cut from the disk. The rectangular prism was then cut into three pieces of 1 cm cubes. The direction of the probe was parallel to the axis of the shoots, where internal hardness (IH) was determined. Each treatment was compressed to 10 mm at a rate of 2 mm/s and the results were expressed in Newtons (N).

### 2.3. Histochemical Localization of Cell Wall Polysaccharides

The periodic acid-Schiff reaction principle was used to specifically determine the polysaccharide components in the cell, as described by Gbalou et al. [14]. Samples of *Z. latifolia* tissues sections were treated with 0.5% periodic acid for approximately 15 min, then treated with Schiff reagent for 30 min, rinsed with rinsing solution three times, observed, and photographed under an optical microscope.

### 2.4. Cell Wall and Polysaccharides Component Preparation

Cell wall polysaccharides were obtained as ethanol-insoluble residues (AIR) using the methods described by Jelle et al. [15]. Briefly, 100 g *Z. latifolia* were ground, extracted with 80% (*v/v*) ethanol, and boiled for 20 min. The residue was re-extracted twice with 80% ethanol and continuously washed with 200 mL 1/1 methanol/chloroform (*v/v*) and 200 mL acetone. The residue was filtered, washed twice with 80% ethanol, filtered again, and dried overnight in a 40 °C oven. The residue composition was expressed in g/100 g FW.

The phenol–sulfuric acid method was applied to determine the content of protopectin and water-soluble pectin, as previously described [8], and calculated using a glucose standard curve.

The lignin and cellulose content was determined by the Klason method, as previously described [16], with slight modifications. Briefly, 100 mg AIR was mixed with 3 mL 72% H_2_SO_4_ (*v/v*) for 3 h, and then diluted with 52.2 mL water to 3% H_2_SO_4_ (*v/v*). These mixtures were then boiled for 2.5 h and then filtered, and the residue was oven dried at 80 °C overnight. After cooling for 1 h at room temperature, Klason lignin was determined gravimetrically, and the results were expressed as the content in 100 mg cell wall substance (mg/100 mg AIR).

### 2.5. Fourier-Transform in Analysis

Fourier-transform infrared (FT-IR) spectroscopy was used to study the structural properties of polysaccharides and reveal characteristic functional groups and stereoisomers. The water extraction–ethanol precipitation method was used to prepare water-extractable polysaccharides using the methods described by Wang et al. [17]. Briefly, 100 mg of AIR was extracted 3 times with distilled water at 90 °C for 3.0 h. The supernatants were combined and concentrated. Then, three times the volume of absolute ethanol was added, and the polysaccharide was precipitated overnight in a refrigerator at 4 °C. The collected precipitate was rotary steamed, dialyzed, concentrated, and lyophilized to obtain water-soluble polysaccharides of *Z. latifolia*. Water-soluble polysaccharides isolated from *Z. latifolia* and stored at different temperatures were characterized via FT-IR spectroscopy. A Nicolet iS50 FT-IR spectrometer was used to scan the FT-IR spectra between 4000 cm^−1^ and 400 cm^−^^1^.

### 2.6. Quality Indexes for Z. latifolia after Storage

The weight loss of *Z. latifolia* was calculated by comparing the weight measured at days 8, 16, 24, and 32 with the original weight (day 0). The colorimeter was used to measure the parameters L*, a*, and b*. The whiteness value (WI) was calculated, as described by Wu et al. [18], using the following formula:

WI = 100 − [(100 − L*)^2^ + a* + b*]^1/2^

The reducing sugar content (RS) was determined, as described previously [19], with some modifications. *Z. latifolia* samples (1 g) were ground into powder in liquid nitrogen, dissolved in 50 mL distilled water, and heated at 80 °C for 30 min. After that, 2 mL supernatant and 1.5 mL DNS reagent were mixed, heated at 95 °C for 5 min, and 3 mL distilled water was added to dilute before the absorbance was measured at 760 nm. Glucose was used to calculate the standard curve, and RS was expressed as a mass fraction (%). Soluble solids (SSC) were ground and filtered from 2 g isolated from the middle section of *Z. latifolia*. A few drops of the filtrate were measured with a digital refractometer to record the °Brix value of each sample.

The relative conductivity, total phenol, and soluble protein were determined as described by Chen et al. [20], with slight modifications. Each group (2 g) of *Z. latifolia* slices (1 mm × 1 mm × 1 μm) was immersed in deionized water for 2 h with shaking. The initial electrolyte leakage (C1) conductivity was measured using a conductivity meter, and then, the solution was boiled for 20 min before the electrolyte leakage (C2) conductivity was measured. The following formula was used to calculate the relative conductivity: (C1/C2) × 100%.

The total phenol content was determined as described by Silva et al. [21], measured using standard gallic acid, and expressed as mg·g^−1^ DW. The soluble protein extract and Coomassie Brilliant Blue were mixed for 2 min, and then the absorbance was recorded at 595 nm. This value was expressed as the mass of soluble protein per gram of fresh weight *Z. latifolia* (mg/g).

### 2.7. Enzyme Activities Related to Cell Wall Polysaccharide Metabolism

An acetic acid-sodium acetate buffer solution (50 mM, pH 5.5) containing 100 mM NaCl, 5% (*w/v*) polyvinylpyrrolidone (PVP), and 2% (*v/v*) mercaptoethanol (50 mM, pH 5.5) was used for enzyme extraction, and was mixed with *Z. latifolia* disrupted into a powder in liquid nitrogen. This mixture was centrifuged, and the supernatant was used to determine the cell wall polysaccharide-metabolizing corresponding enzyme activity. This enzyme activity measurement was slightly modified according to Chen et al. [6].

Pectin methyl esterase (PME) was assayed in a reaction mixture containing 0.5% (*w/v*) pectin, bromothymol blue (0.01%), and crude enzyme solution and recorded at 620 nm. PME activity was defined as the change in absorbance at 0.01/min, and was expressed as U kg^−1^ FW. PG activity was assayed according to Chen et al. [22]. An aliquot of 0.1 mL crude enzyme solution was mixed with 0.4 mL 1% polygalacturonic acid (*w/v*) and mixed at 37 °C for 30 min. After cooling, it was mixed with 2.5 mL 3,5-dinitrosalicylic acid reagent, the absorbance was measured at 540 nm.

Polygalacturonase (PG) activity was expressed as the ability to produce 1 mg galacturonic acid per minute. β- Galactosidase (β-Gal) activity was assayed using the method described by Lin et al. [11]. The mixture containing 16 M p-nitrophenyl-β-d-galactopyranoside and the crude enzyme solution was shaken in a 37 °C water bath for 90 min before the reaction was stopped with 2 mL 1 M Na_2_CO_3_. The absorbance of this solution was measured at 400 nm. The β-Gal activity was the ability to produce 1 mg PNP per minute and expressed as U kg^−1^ FW.

Cellulase (Cx) activity was determined as described by Chen et al. [22]. The reaction mixture contained 0.1 mL crude enzyme solution and 1.5 mL sodium carboxymethyl cellulose (CMC) (1%, *w/v*). An enzyme solution inactivated in a boiling water bath was used as a control. The enzyme mixtures were incubated at 37 °C for 1 h. Following rapid cooling, the absorbance was measured at 540 nm. One unit of the enzyme was defined as the amount of enzyme required to produce 1 μmol of glucose in 1 h.

### 2.8. Enzyme Activities Related to Lignin Metabolism

Polyphenol oxidase (PPO) was assayed in a reaction mixture containing a crude enzyme solution, 100 mM sodium acetate buffer, and 50 mM catechol. A unit of PPO activity was defined as the change in absorbance of 0.01 at 420 nm per gram of fresh weight of *Z. latifolia* per minute. Peroxidase (POD) was assayed in a reaction mixture of 25 mM guaiacol, 0.5 M hydrogen peroxide, and crude enzyme. One POD activity unit was defined as the 0.01 absorbance change at 470 nm per gram of fresh weight *Z. latifolia* per minute. Laccase (LAC) was assayed in a reaction mixture containing ABTS, a crude enzyme solution, and 100 mM PBS. One unit of laccase activity was defined as the amount of enzyme that produced a 0.01/min change in absorbance at 436 nm.

### 2.9. Data Statistics and Analysis

All experiments were repeated three times. One-way ANOVA with Duncan’s multiple comparisons was analyzed. Excel 2016 was used to collate and analyze the collected data. SPSS Statistics 20 was used to perform Duncan’s analysis of variance, and Pearson’s chi-square test. Illustrations were constructed with Prism v8.0.2, and R software was used to generate the correlation matrix and principal component analysis (PCA) graphs.

## 3. Results and Discussion

### 3.1. Change of Appearance and Hardness of Harvested Z. latifolia

**Figure 1:**
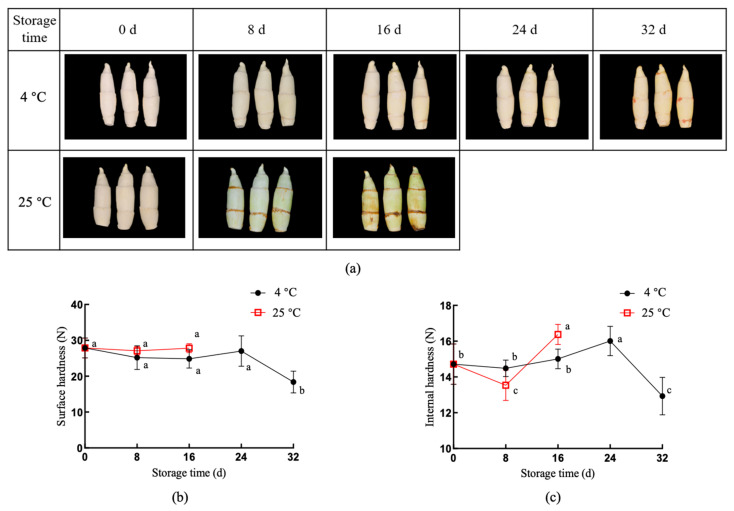
Changes in the hardness of *Z. latifolia* during storage at different temperatures. (**a**) Images of *Z. latifolia* shoots stored at 4 °C and 25 °C over time indicates changes in physical traits. (**b**) Measurement of surface hardness and (**c**) internal hardness of *Z. latifolia* shoots stored at 4 °C and 25 °C. Each treatment was repeated 12 times. The bars showed the standard deviations of the mean and the letters indicated statistically different for each group by Duncan’s multiple ranges (*p* < 0.05) (*n* = 12). Different letters in (**b**,**c**) indicate the significance of differences between groups over time.

We first analyzed the characteristics of harvested *Z. latifolia*. The visual appearance of the samples at 0, 8, and 16 days at 25 °C and 0, 8, 16, 24, and 32 days at 4 °C is shown in Figure 1a. When stored at 25 °C for 16 days, the skin of *Z. latifolia* shoots partially browned and the appearance turned green, while low temperature inhibited the increase in greenness incidence and maintained the whiteness of *Z. latifolia*. When stored at 4 °C for 32 days, the wilting and skin browning in the shoots were less than those stored at room temperature.

For *Z. latifolia* stored at 25 °C, the surface hardness (SH) did not change significantly between days 0 and 16 (Figure 1b). Further comparison showed that there was no significant change in the surface hardness after storage at 4 °C (0–24 days), and then gradually decreased (*p* < 0.05). As shown in Figure 1c, compared with storage at 4 °C, the internal hardness (IH) of *Z. latifolia* stored at 25 °C was lower on the 0 day–8 days. Statistical analysis showed (Figure 1c) that the internal hardness of *Z. latifolia* at 4 °C changed little from day 0 to day 16, and increased gradually from storage day 16 and day 24, followed by a gradual decrease.

### 3.2. Cell Wall Polysaccharide Staining Results

**Figure 2:**
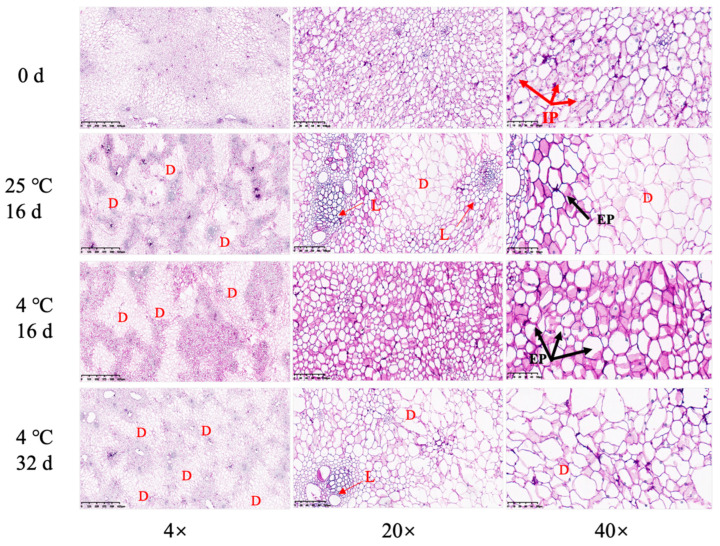
Polysaccharide staining of *Z. latifolia* cell walls using PAS during storage at different temperatures. D, polysaccharide degradation; L, lignification; IP, intracellular polysaccharide; EP, extracellular polysaccharide.

Periodic acid-schiff stain (PAS) was used to stain the cellulosic cell walls purple–red, and lignin in the cell walls was dyed blue–green. For *Z. latifolia* stored at 25 °C, cell wall lignification appeared at 16 days, and local cell wall polysaccharides were depolymerized and solubilized. For *Z. latifolia* stored at 4 °C, less lignified *Z. latifolia* cell walls were observed (Figure 2, red arrows), polysaccharides were unevenly distributed, and cellulose cell walls were locally accumulated. There was low lignification and high degradation of polysaccharides at 4 °C, indicating that low-temperature storage delayed the lignification process of *Z. latifolia* shoots.

Extracellular polysaccharides (EP) are one of the main components of biofilm matrices. The black arrows in Figure 2 indicate where exopolysaccharide production was observed in the stored *Z. latifolia* shoots. The cell wall is mainly composed of structural polysaccharides such as cellulose and pectin, as well as lignin, and cell wall components play an important role in fruit texture [23]. Our results showed that the cell wall was prone to be lignified under 25 °C storage conditions (lignin accumulation), while more cellulose was accumulated at 4 °C.

### 3.3. Cell Wall Preparation and Polysaccharide Components

**Figure 3:**
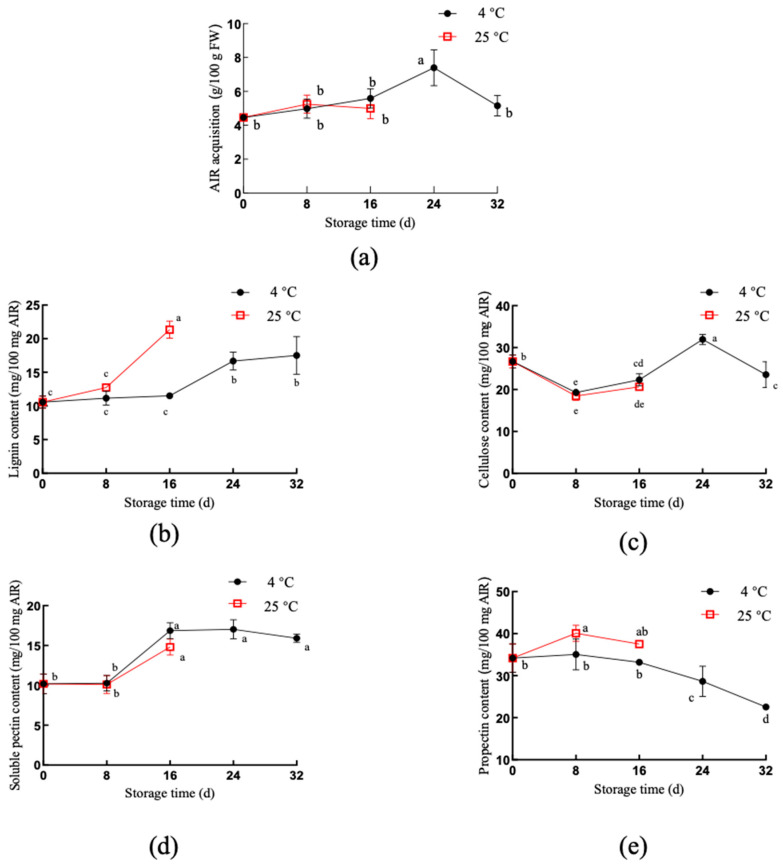
Changes of cell wall polysaccharide composition in *Z. latifolia* during storage at different temperatures. (**a**) Alcohol insoluble matter (AIR), (**b**) lignin content (LIG), (**c**) cellulose content (CEL), (**d**) soluble pectin content (SPC), and (**e**) propectin content (PROP) were measured from samples stored at either 4 °C or 25 °C. Each value was expressed as mean ± standard error (*n* = 3). Different letters indicate a significant difference between the different storage times (*p* < 0.01).

The content and structure of cell wall polysaccharides play vital roles in fruit texture [24]. The depolymerization, solubilization, desertification, and loss of side chains of pectin molecules make the fruit soft [25]. We found that the composition of the *Z. latifolia* cell wall changed under different temperature storage conditions (Figure 3). When stored at 25 °C, alcohol-insoluble residues (AIR) showed less pronounced changes over time (Figure 3a). During storage at 4 °C, the AIR reached its maximum on day 24 (7.40 g/100 g FW), and then decreased. Comparisons of the relative contribution of the four major components to the overall cell wall compositions (Figure 3b–e) suggested that they may help to distinguish between accelerated deposition of a certain component and coordinated cell wall metabolism. During storage at 25 °C, the lignin (LIG) content of *Z. latifolia* was significantly increased (*p* < 0.01), while the cellulose (CEL) content was decreased (*p* < 0.01); the content was 21.04 mg/100 mg AIR and 21.35 mg/100 mg AIR on day 16, which increased by 1.02 times and decreased by 2.38 times, respectively, from the day 0 measurements (Figure 3b,c). We also found that 4 °C storage partially inhibited lignification, at least during the early storage period, and then slightly increased from storage day 16 to day 32. Cellulose decreased rapidly during the first eight days of 4 °C storage, then slightly increased from day 8 to day 24, and then gradually increased.

When stored at 4 °C, *Z. latifolia* had higher levels of cellulose than those stored at 25 °C (Figure 3c). When stored at 4 °C, cellulose increased during the storage period of 8–14 days, while protopectin content (PROP) decreased; the internal hardness of *Z. latifolia* initially increased before 24 days, then decrease thereafter (*p* < 0.01), which may be attributed to the increase in cellulose content during storage (8–14 days). During the later storage period (24–32 days), the hardness decreased due to a reduction of protopectin and cellulose contents. When stored at 25 °C, the contents of lignin and protopectin increased during the storage period 8 days to 16 days (*p* < 0.01), which was accompanied by an increase in hardness.

Further detection of cell wall polysaccharide composition and correlation analysis (Figure 5) showed that the internal hardness was highly correlated with changes in protopectin content (r^2^ = 0.95; *p* < 0.01). This was attributed to the hydrolysis of protopectin into soluble pectin, as the cell wall became thinner and separated from each other, which led to changes in its texture [11]. However, protopectin and water-soluble pectin do not necessarily have a trade-off relationship. Ren et al. [26] found that protopectin and soluble pectin increased in the process of softening after harvest, which was consistent with the change in water glutinous pectin polysaccharide at 25 °C in this study. Moreover, the cellulose content of the cell wall was closely related to the texture change. Bunsiri et al. [27] studied the significant changes in the cellulose content and moisture content of mangosteen peel, while the increase in peel hardness was mainly due to the cell wall cellulose and woodiness. In our study, cellulose, and lignin content were positively correlated (r^2^ = 0.95; *p* < 0.01), and surface hardness was highly positively correlated (r^2^ = 0.69; *p* < 0.01) with cellulose and lignin content, indicating that as the important components of cell wall components, cellulose and lignin exert various effects in the postharvest texture changes.

### 3.4. Infrared Spectroscopy of Water-Soluble Polysaccharides in Z. latifolia Stored at Different Temperatures

**Figure 4:**
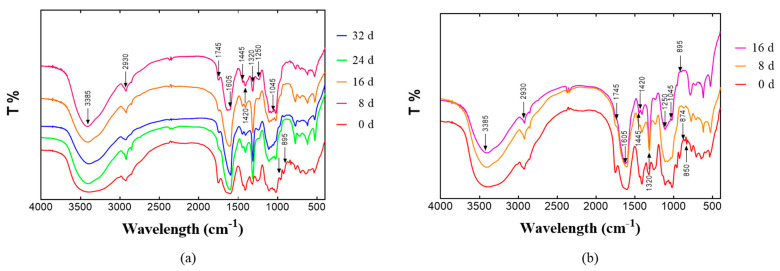
FT-IR spectra of water-soluble polysaccharides isolated from *Z. latifolia* at different storage periods. (**a**) Storage at 4 ℃, (**b**) storage at 25 °C.

Fourier transform infrared (FT-IR) spectra of the water-soluble polysaccharides in *Z. latifolia* stored at different temperatures (Figure 4a,b) were typical for polysaccharides, and within the range of 4000–400 cm^−1^. The abscissa is the transmittance (T%). The FT-IR spectra showed a broad hydroxyl peak around 3400 cm^−1^ and weak peaks of single bonds around 2920 cm^−1^ and 1420 cm^−1^, which were characteristic absorption peaks of polysaccharides [28]. The peak at 1320 cm^−1^ was related to aromatic compounds [29], while the peaks representing esterified carboxyl groups and free carboxyl groups in pectin were observed at 1745 cm^−1^ and 1605 cm^−1^, which indicated that the degree of methyl esterification of water-soluble polysaccharides was reduced and that aromatic compounds may be produced during storage. These results indicated that lowering in the esterification of water-soluble polysaccharides occurred more at 25 °C, compared to that at 4 °C storage. The carboxylic groups of de-esterified pectins were cross-linked by Ca^2+^ in the middle lamella of the cell wall, which strengthened the cell wall structure of *Z. latifolia* stored at 25 °C. The weak peak at 1250 cm^−1^ was due to the stretching vibration of S-O, which indicated that the polysaccharide contained sulfate [17]. The absorption at 1000–1200 cm^−1^ was attributed to the overlap of the ring vibration with the stretching vibration of the C-OH side group and the vibration of the C-O-C glycoside band, which indicated the presence of sugar in the form of pyranose [17]. The absorption at 951 cm^−1^ indicated the presence of β-type glycosidic bonds [28,30]. The *Z. latifolia* stored at different temperatures produced polysaccharides with reduced peak intensities at 926 cm^−1^ and 850 cm^−1^, which were characteristic of 3,6-anhydrogalactose and α-glycosidic bonds, respectively [31].

### 3.5. Quality Changes of Z. latifolia during Storage

The changes in the quality index of *Z. latifolia* during storage at 4 °C and 25 °C are shown in Table 1. During storage at 4 °C, the reducing sugar (RS), soluble solid (SST), and whiteness values (WI) of *Z. latifolia* continued to decrease. After 32 days of storage, these factors decreased 0.77, 0.52, and 0.15 times, respectively, when compared with fresh *Z. latifolia*. The reducing sugar content, soluble solid content, and whiteness value of *Z. latifolia* stored at 25 °C were significantly reduced after 8 days of storage (*p* < 0.05). *Z. latifolia* is rich in a variety of polyphenols, and their oxidation products are the precursors of synthetic lignin, which is the main substrate for enzymatic browning [32]. Phenolic compounds are bound via ester and ether linkages to the structural components of the cell walls [33]. Our previous study analyzed the detailed profiles of phenolics in *Z. latifolia*, which including butyl isobutyl phthalate vanillin, ferulic acid methyl ester, chlorogenic acid methyl ester, 1-O-Feruloylquinic acid, and p-Coumaric acid [34]. Wang et al. found that chlorogenic acid, neochlorogenic acid, and protocatechuic acid were the dominating phenolic compounds in loquat pulp, and these three compounds increased in the early stage of storage, and then decreased in the middle stage [33]. In the present study, the total phenol content (TP) increased from 0 day to 16 days, which was followed by a slight decrease during storage at 4 °C. When stored for 16 days, the maximum total phenol content reached 2.63 mg/g FW. The total phenol content of *Z. latifolia* stored at 25 °C increased from 0 day to 8 days and then sharply decreased. Soluble protein (SP) and TP showed the same trend. When stored for 8 d at 25 °C, the highest total phenol content was 3.40 mg/g. Both weight loss (WL) and relative conductivity (RC) continued to increase. The whiteness value dropped below 74, and microorganisms began to appear on the surface. These results may be reflected by the postharvest physiological metabolism of *Z. latifolia*.

### 3.6. Changes in Cell Wall Metabolism Corresponding Enzyme Activity in Z. latifolia during Storage

As shown in Table 2, during the storage period, the Cx activity of *Z. latifolia* continued to increase overall when stored at 4 °C. However, when stored at 25 °C, Cx activity increased rapidly during days 0–8 and then decreased during days 8–16. On day 16, the Cx, PG and β-gal activity of *Z. latifolia* stored at 25 °C was significantly lower than that of the 4 °C storage group (*p* < 0.01). PG and PME activities were linked to ethylene production and softening [26]. The activities of PG and PME gradually increased with increasing storage time. The activity of PG increased quickly in storage during days 0–16 and then dropped rapidly after 16 d of storage, and the activity of PME reached its peak at a relatively late storage time (24 days). Similar to PME, the β-gal activity of *Z. latifolia* increased throughout the storage process and increased rapidly on days 8, when stored at 25 °C.

The postharvest *Z. latifolia* hardening was related to lignification, which was mainly attributed to the decreased activities of lignin biosynthesis-related enzymes, and induced activation of antioxidant-corresponding enzymes [3]. POD catalyzes the oxidation of polyphenols and is closely related to lignin synthesis [35]. During the storage process, the activity of POD continued to increase, whereas the activity of PPO remained decreased. The PPO activity of *Z. latifolia* stored at 25 °C was significantly higher than that stored at 4 °C (*p* < 0.01). Laccase (LAC) catalyzes the oxidation of a wide range of phenolic substrates, which is related to the synthesis of lignin [36]. The laccase activity of *Z. latifolia* stored at 4 °C showed a downward trend during the storage process and increased rapidly during days 0–8 at 4 °C.

### 3.7. Correlation Analysis

**Figure 5:**
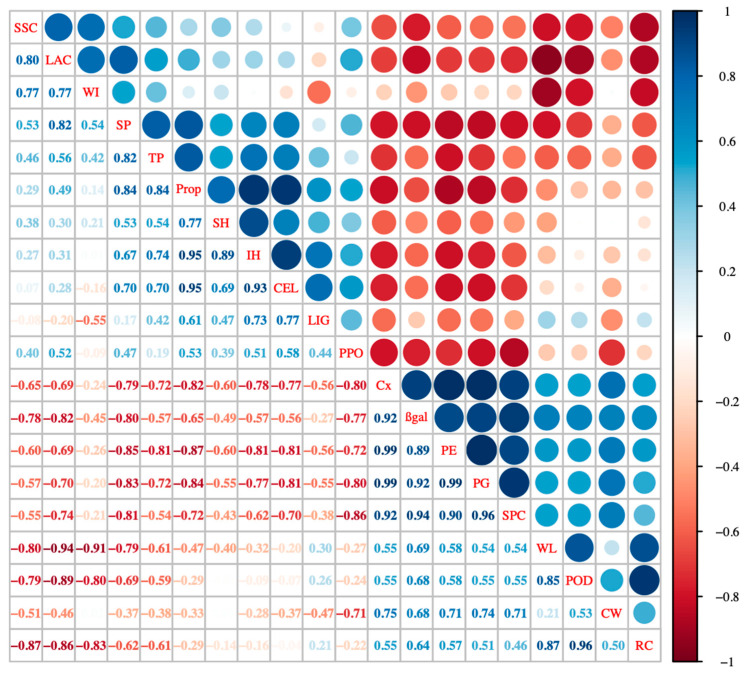
Pearson’s correlation matrix between texture index, cell wall components and relative enzymes. The correlation coefficients are proportional to the magnitude of the value and the intensity of the color. Blue indicates positive correlation, and negative correlation is shown in red (*p* < 0.01). (*n* = 3). RS, reducing sugar content; TTS, soluble solid content; SPC, soluble pectin; SP, soluble protein; PPO, polyphenol oxidase; PROP; protopectin; β-gal, β-half lactosidase; SH, surface hardness; IH, internal hardness; WL, weight loss rate; RC, relative conductivity; POD, peroxidase; LAC, laccase activity; WI, whiteness value; CEL, cellulose content; LIG, lignin content; Cx, cellulase; CW, cell wall content; PME, pectin methyl esterase; PG, polygalacturonase; TP, total polyphenol content.

The depolymerization or deposition of polysaccharides in the cell wall can reduce the texture characteristics of *Z. latifolia*, for example, an increase in the lignocellulosic thickens the cell wall and increases the hardness [37]. Correlation analysis showed that hardness was related to cell wall metabolic corresponding enzyme activity (β-gal, PG, Cx, and PE) and cell wall composition (prop, SPC, SP) (*p* < 0.01), with the strongest correlation being between protopectin and pectin methylesterase. Therefore, the texture of *Z. latifolia* was related to the content of pectin methylesterase and protopectin. IH and CEL contents were significantly positive correlated (r^2^ = 0.93; *p* < 0.01). Other studies have reported similar results on the effects of cell wall composition on fruit hardness during the postharvest ripening process [10,38]. Weight loss rate and hardness were negatively correlated, with a correlation coefficient of −0.4 (*p* < 0.01). A previous study reported that the moisture content in the pericarp of *Annona squamosa* gradually decreased during the storage process. Besides, the freedom degree of moisture was associated with a softer texture and cell wall degradation [39]. As shown in Figure 5, prop and SPC content were significantly negatively correlated, with a correlation coefficient of −0.72 (*p* < 0.01). In addition, there was a significant positive correlation between SPC and cell wall metabolic enzymes (β-gal, PG, Cx, and PE) and CW (*p* < 0.01), which indicated that changes in the texture of *Z. latifolia* were accompanied by an increase in soluble pectin and cell wall enzymatic degradation of polysaccharides. The RS, SSC, WI, SP, TP, LAC, and PPO were negatively correlated with the rate of weight loss, while CW, RC, SPC, and POD were positively correlated with it. These results indicated that laccase and POD play important roles in the postharvest water loss and shrinkage in *Z. latifolia*. POD catalyzed the oxidation of phenols to quinones, and there was a certain negative correlation between them (r^2^ = −0.59; *p* < 0.01). PPO was related to tissue browning; however, it showed little correlation with whiteness values in *Z. latifolia* during storage. Wen et al. reported that all chlorophyll contents of *Z. latifolia* increased throughout the storage period, which reduced the whiteness value [3]. In our study, we also found that *Z. latifolia* stored at 25 °C tended to be green in color (Figure 1a).

### 3.8. Relevance Analysis

**Figure 6:**
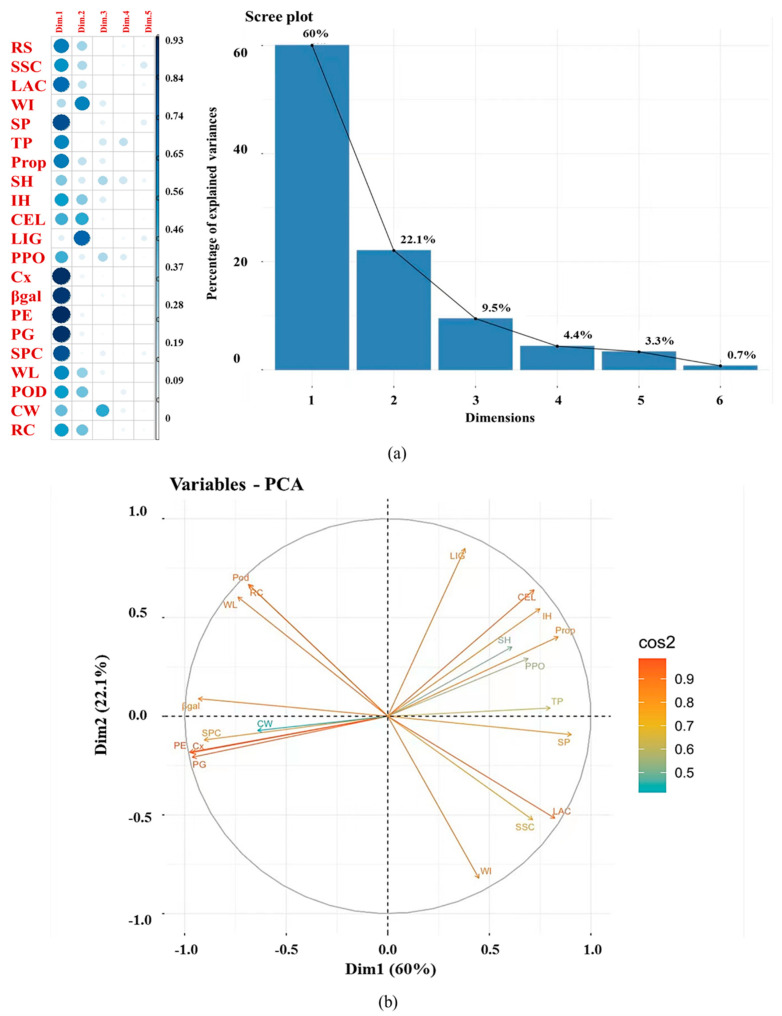
Principal component analysis of the quality parameters, chemical composition, and metabolic enzyme activities in *Z. latifolia* stored at 4 °C and 25 °C. (**a**) Principal component analysis scree plot (*n* = 3), (**b**) factor loading plots (*n* = 3). The X-axis and Y-axis correspond to Principal component 1 (Dim1) and Principal component 2 (Dim2), respectively. RS, reducing sugar content; TTS, soluble solid content; SPC, soluble pectin; SP, soluble protein; PPO, polyphenol oxidase; PROP; protopectin; β-gal, β-half lactosidase; SH, surface hardness; IH, internal hardness; WL, weight loss rate; RC, relative conductivity; POD, peroxidase; LAC, laccase activity; WI, whiteness value; CEL, cellulose content; LIG, lignin content; Cx, cellulase; CW, cell wall content; PME, pectin methyl esterase; PG, polygalacturonase; TP, total polyphenol content.

PCA was used to evaluate changes in *Z. latifolia* quality indicators, cell wall components, and metabolic enzyme activities during storage at 4 °C and 25 °C. As shown in Figure 6, three principal components were identified. Principal component 1 (Dim1) had the strongest correlation, which included quality indicators (hardness, SSC, and WL), SP, TP, LAC, Prop, Cx, β-gal, PE, PG, and SPC. Principal component 2 (Dim2) included LIG, WI, and CEL. Principal component 3 (Dim3) included only CW (*n* = 3). Dim1 indicated the direction of the greatest change in the dataset. Dim1 and Dim2 accounted for 82.6% of the total variability of the original data, which was used to assess postharvest metamorphism. The dispersion of Dim1 and Dim2 and the load of each variable are shown in Figure 4. Negatively correlated variables are positioned on opposite sides of the plot origin, while positively correlated variables are grouped together. During storage, cell wall metabolic enzymes that contributed to the changes in cell wall composition included Cx, β-gal PE, PG, POD, PPO, and laccase. Through PCA analysis, variables that were positioned farther from the center of the plot were more important for the first components (Dim1). The cos2 values were used to estimate the quality of the representation. The closer a variable was to the circle of correlations, the better its representation on the factor map (and the more important it is to interpret these components). A high cos2 is displayed in red and a low cos2 in blue (Figure 6b). These results indicate that cell wall metabolic enzymes (Cx, PE, PG, and β-gal) were distal from the center of the plot and were involved in the changes in cell wall composition during the postharvest storage of *Z. latifolia*.

Lin et al. found that the activity of Cx and β-gal played important roles in the degradation of pectic polymers during softening, especially in the late stage of softening, which was consistent with our results [11]. In previous studies, the combined effect of PE and PG was reported to degrade pectin substances, and before PG was activated, the pectin chain must be demethylated and esterified by PME, which makes it more susceptible to the effect of PG. The highest activity of PE appeared earlier than that of PG [35]. An early increase in PME activity and a delayed but significant increase in PG activity was observed in winter jujube fruit during storage [5]. In our study, the increase in *Z. latifolia* hardness could be attributed to the highest activity of PG appearing earlier than that of PE. In addition, there was a significant negative correlation between PE and *Z. latifolia* internal hardness (r^2^ = −0.81; *p* < 0.01), which may be due to the low activity of PE in the early storage period that limited the hydrolysis of PG on pectin and the hydrolysis rate of protopectin changes [40]. The hardness continued to increase in the early stages of storage. PPO-oxidized polyphenols, PPO, and laccase are responsible for the polymerization and decomposition of lignin [41]. Low-temperature storage delayed the lignification process by inhibiting the activity of lignin synthase, which was consistent with the results of Qi et al. [13]. Enzymes (Cx, PE, PG, and β-gal) and SPC were grouped with CW, away from the center of the plot, which indicated that the first latent variable (Dim1) was related to changes in SPC and cell wall metabolic corresponding enzyme activity (Figure 6a). Similarly, CEL, Prop, and LIG were far away from the center of the plot, in addition to hardness, which indicated that the change in cell wall composition contributed to the change in *Z. latifolia* texture during storage.

Different enzymes are involved in CW degradation, such as PME, PG, Cx, and β-gal, which all act in an interdependent manner. PME and PG are key enzymes associated with the demethylation of pectin and hydrolysis of pectate by cleaving the α-(1,4)-glycosidic bonds, respectively [8]. Besides, cellulose is widely regarded to cause the degradation of cellulose matrix in the cell walls. Ali et al. found that tragacanth gum application suppressed PG, PME, and Cx enzymes activities, thereby suppressing the softening of apricot fruits [42]. Our results showed that low-temperature storage delayed degrading enzyme activities, leading to an increase in cell wall components and polysaccharide production which, in turn, led to the promotion of fibrosis, delayed lignification, increased hardness in the early storage stage, and accelerated decomposition of pectin polymers in the later stage of storage.

These results indicate that the deterioration process of *Z. latifolia* was regulated by cell wall polysaccharide metabolism. However, the molecular mechanism underlying the changes in the texture of *Z. latifolia*, caused by storage at different temperatures, was still unclear. Lin et al. found that the expression levels of cell wall polysaccharides degradation-related genes (*DlXET*, *DlPE*, *DlPG*, *Dlβ-Gal*, and *DlCx*) played important roles in accelerating the decomposition of longan pulp cell wall polysaccharides [11]. Therefore, it is necessary to combine metabolomics and transcriptomics analysis results for elucidating the molecular mechanism behind polysaccharide metabolism in the cell wall of *Z. latifolia*.

## 4. Conclusions

In this study, postharvest metamorphism in *Z. latifolia* was monitored at 4 °C and 25 °C. The quality indicators, cell wall components, and cell wall metabolic enzyme activities of *Z. latifolia* were evaluated. Statistical analysis showed that hardness was related to cell wall composition (prop, SPC, and SP) and corresponding enzyme activity, while water loss and shrinkage were accelerated by the increase in corresponding enzyme activity (LAC and PPO) and reducing sugars. The degradation of cell wall polysaccharides, especially pectin polysaccharides, destroys the network structure of the cell wall, which results in changes in the texture of postharvest *Z. latifolia*. During storage, cell-wall-modifying enzymes, such as PG, PME, and Cx, played important roles in the degradation of cell wall polysaccharides, and protopectin was degraded by coordinated PME enzyme pathways (r^2^ = −0.81; *p* < 0.01). PCA analysis showed that the first latent variable was related to changes in SPC and cell wall metabolic corresponding enzyme activity, while the change in cell wall composition contributed to the change in texture. These results improve our understanding of the underlying mechanism of *Z. latifolia* deterioration during storage, and may provide new insights for the development of new methods for its preservation.

## Figures and Tables

**Table 1:** Changes in quality of *Z. latifolia* at different storage temperatures.

Temperature (℃)	Storage Time (days)	Reducing Sugar (%)	Total Phenol (mg/100 g)	Soluble Solid Content (%)	Whiteness Value (%)	Weightlessness Rate (%)	Relative Conductivity (%)	Soluble Protein (mg/g)
4 °C	0	22.47 ± 0.64 ^a^	2.12 ± 0.07 ^b^	6.10 ± 0.20 ^a^	87.00 ± 0.53 ^a^	0.00 ^d^	14.37 ± 1.40 ^b^	3.03 ± 0.41 ^b^
	8	17.55 ± 0.71 ^a^	2.42 ± 0.12 ^ab^	5.50 ± 0.61 ^a^	86.90 ± 0.91 ^a^	1.72 ± 0.38 ^c^	16.16 ± 0.43 ^b^	3.15 ± 0.07 ^a^
	16	10.06 ± 0.37 ^b^	2.63 ± 0.19 ^a^	3.83 ± 0.15 ^b^	85.99 ± 0.67 ^ab^	2.72 ± 1.22 ^c^	15.41 ± 0.27 ^b^	3.11 ± 0.02 ^a^
	24	6.37 ± 0.45 ^bc^	2.47 ± 0.05 ^ab^	2.87 ± 0.50 ^bc^	81.59 ± 1.60 ^b^	5.11 ± 2.27 ^b^	20.74 ± 0.19 ^a^	2.61 ± 0.10 ^c^
	32	4.79 ± 0.51 ^c^	2.19 ± 0.07 ^ab^	2.33 ± 0.12 ^c^	76.89 ± 2.23 ^c^	8.00 ± 3.42 ^a^	19.40 ± 0.95 ^a^	2.95 ± 0.21 ^b^
25 °C	0	22.47 ± 0.64 ^a^	2.12 ± 0.07 ^b^	6.10 ± 0.20 ^a^	87.00 ± 0.53 ^a^	0.00 ^c^	14.37 ± 1.40 ^c^	3.04 ± 0.41 ^a^
	8	10.28 ± 0.36 ^b^	3.40 ± 0.27 ^a^	2.67 ± 0.42 ^b^	81.89 ± 1.15 ^b^	2.35 ± 1.05 ^b^	17.71 ± 0.36 ^b^	3.22 ± 0.02 ^a^
	16	5.45 ± 0.42 ^c^	2.15 ± 0.01 ^b^	2.93 ± 0.81 ^b^	74.40 ± 2.19 ^c^	7.70 ± 2.80 ^a^	20.91 ± 0.86 ^a^	3.00 ± 0.15 ^a^

Values are expressed as mean ± SD (*n* = 3). One-way ANOVA was analyzed (*p* < 0.05). Lowercase letters among different stages within each group indicate significantly different values according to Duncan’s multiple comparison test. Different letters above groups represent significant differences, shared letters represent no significant differences.

**Table 2:** Changes in enzymes related to cell wall metabolism of *Z. latifolia* during storage.

Temperature (℃)	Storage Time (d)	Cx (U/g)	β-gal (U/g)	PG (U/g)	PME (U/g)	Lac (U/g)	PPO (U/g)	POD (U/g)
4 °C	0	29.59 ± 0.86 ^c^	9.79 ± 0.27 ^b^	5.49 ± 0.15 ^c^	6.26 ± 1.52 ^c^	12.92 ± 1.21 ^a^	7.98 ± 0.35 ^a^	12.27 ± 3.27 ^c^
	8	42.90 ± 2.83 ^b^	9.74 ± 0.17 ^b^	5.01 ± 0.13 ^c^	6.47 ± 0.96 ^bc^	11.42 ± 0.47 ^a^	1.89 ± 0.12 ^c^	13.65 ± 2.83 ^c^
	16	79.00 ± 2.0 ^a^	23.73 ± 0.06 ^a^	10.5 ± 0.21 ^b^	7.14 ± 0.43 ^abc^	5.21 ± 0.52 ^b^	1.46 ± 0.31 ^c^	17.95 ± 1.90 ^b^
	24	41.79 ± 1.09 ^b^	23.82 ± 1.59 ^a^	13.31 ± 0.26 ^a^	10.06 ± 0.75 ^a^	5.57 ± 1.27 ^b^	2.08 ± 1.03 ^c^	40.99 ± 3.77 ^a^
	32	10.83 ± 1.69 ^d^	22.61 ± 0.83 ^a^	10.26 ± 0.54 ^b^	9.39 ± 2.09 ^ab^	4.76 ± 0.51 ^b^	3.90 ± 0.48 ^b^	32.44 ± 5.10 ^ab^
25 °C	0	29.59 ± 0.86 ^b^	9.79 ± 0.27 ^c^	5.49 ± 0.15 ^b^	6.26 ± 1.52 ^a^	12.92 ± 1.21 ^a^	7.98 ± 0.35 ^a^	12.27 ± 3.27 ^c^
	8	88.73 ± 1.29 ^a^	23.77 ± 0.16 ^a^	6.35 ± 0.71 ^c^	6.20 ± 0.21 ^a^	19.19 ± 0.41 ^a^	6.71 ± 0.96 ^a^	21.32 ± 3.96 ^b^
	16	59.83 ± 1.55 ^b^	22.54 ± 0.69 ^b^	15.35 ± 0.90 ^a^	7.44 ± 1.13 ^a^	12.18 ± 0.98 ^b^	6.39 ± 0.26 ^a^	43.41 ± 4.94 ^a^

Values are expressed as mean ± SD (*n* = 3) within each column. Lowercase letters represent significant differences within groups according to Duncan’s multiple rage tests (*p* < 0.01). Cx, cellulase activity; β-gal, β-galactosidase activity; PG, polygalacturonase actnivity; PME, pectin methylesterase; PPO, polyphenol oxidase; POD, peroxidase.

## Data Availability

The data presented in this study are available on request from the corresponding author.

## References

[1] Ye C., He C., Zhang B., Wang L., Wang L. (2020). Inhibition of lignification of *Zizania latifolia* with radio frequency treatments during postharvest. BMC Chem..

[2] Yan N., Du Y., Liu X., Cheng C., John S., Zhang H., Liu Y., Zhang Z. (2018). Morphological characteristics, nutrients, and bioactive compounds of *Zizania latifolia*, and health benefits of its seeds. Molecules.

[3] Wen B., Cheng Z., Hu Y., Chalermchai W., Suriyan S. (2019). Ultraviolet-C treatment maintains physicochemical quality of water bamboo (*Zizania latifolia*) shoots during postharvest storage. Postharvest Biol. Technol..

[4] Li Q., Xu R., Fang Q., Yuan Y., Cao J., Jiang W. (2020). Analyses of microstructure and cell wall polysaccharides of flesh tissues provide insights into cultivar difference in mealy patterns developed in apple fruit. Food Chem..

[5] Zhao Y., Zhu X., Hou Y., Wang X., Li X. (2019). Effects of nitric oxide fumigation treatment on retarding cell wall degradation and delaying softening of winter jujube (*Ziziphus jujuba* Mill. cv. Dongzao) fruit during storage. Postharvest Biol. Technol..

[6] Fan X., Jiang W., Gong H., Yang Y., Zhang A., Liu H., Cao J., Guo F., Cui K. (2019). Cell wall polysaccharides degradation and ultrastructure modification of apricot during storage at a near freezing temperature. Food Chem..

[7] Chen Y., Zhang S., Lin H., Lu W., Wang H., Chen Y., Lin Y., Fan Z. (2021). The role of cell wall polysaccharides disassembly in *Lasiodiplodia theobromae*-induced disease occurrence and softening of fresh longan fruit. Food Chem..

[8] Wang H., Wang J., Mujumdar A.S., Jin X., Liu Z.-L., Zhang Y., Xiao H.-W. (2021). Effects of postharvest ripening on physicochemical properties, microstructure, cell wall polysaccharides contents (pectin, hemicellulose, cellulose) and nanostructure of kiwifruit (*Actinidia deliciosa*). Food Hydrocolloid..

[9] Zhang J., Yin X.-R., Li H., Xu M., Zhang M.-X., Li S.-J., Liu X.-F., Shi Y.-N., Grierson D., Chen K.-S. (2020). Ethylene response factor39–MYB8 complex regulates low-temperature-induced lignification of loquat fruit. J. Exp. Bot..

[10] Cui K., Yang L., Shu C., Liu J., Zhu Z., Yang Z., Zhu X., Jiang W. (2021). Near freezing temperature storage alleviates cell wall polysaccharide degradation and softening of apricot (*Prunus armeniaca* L.) fruit after simulated transport vibration. Sci. Hortic..

[11] Lin Y., Lin H., Wang H., Lin M., Chen Y., Fan Z., Hung Y., Lin Y. (2020). Effects of hydrogen peroxide treatment on pulp breakdown, softening, and cell wall polysaccharide metabolism in fresh longan fruit. Carbohyd. Polym..

[12] Song L., Gao H., Chen W., Chen H., Mao J., Zhou Y., Duan X., Joyce D.-C. (2011). The role of 1-methylcyclopropene in lignification and expansin gene expression in peeled water bamboo shoot (*Zizania caduciflora* L.). J. Sci. Food Agric..

[13] Qi X., Ji Z., Lin C., Li S., Liu J., Kan J., Zhang M., Jin C., Qian C. (2020). Nitric oxide alleviates lignification and softening of water bamboo (*Zizania latifolia*) shoots during postharvest storage. Food Chem..

[14] Randrianjatovo-Gbalou I., Girbal-Neuhauser E., Marcato-Romain C.-E. (2016). Quantification of biofilm exopolysaccharides using an in-situ assay with periodic acid-Schiff reagent. Anal. Biochem..

[15] Jelle V.-A., Tom B., Victor D.-S., Sophie D., Van L.-A.-M., Hendrickx M.-E. (2021). The structure and composition of extracted pectin and residual cell wall material from processing tomato: The role of a stepwise approach versus high-pressure homogenization-facilitated acid extraction. Foods.

[16] Song L., Chen H., Gao H., Fang X., Mu H., Yuan Y., Yang Q., Jiang Y. (2013). Combined modified atmosphere packaging and low temperature storage delay lignification and improve the defense response of minimally processed water bamboo shoot. Chem. Cent. J..

[17] Wang M., Zhao S., Zhu P., Nie C., Ma S., Wang N., Du X., Zhou Y. (2018). Purification, characterization and immunomodulatory activity of water extractable polysaccharides from the swollen culms of *Zizania latifolia*. Int. J. Biol. Macromol..

[18] Wu W., Gao H., Chen H., Fang X., Han Q., Zhong Q. (2018). Combined effects of aqueous chlorine dioxide and ultrasonic treatments on shelf-life and nutritional quality of bok choy (*Brassica chinensis*). LWT Food Sci. Technol..

[19] Nam H.-A., Ramakrishnan S., Kwon J.-H. (2019). Effects of electron-beam irradiation on the quality characteristics of mandarin oranges (*Citrus unshiu* (Swingle) Marcov) during storage. Food Chem..

[20] Chen H., Cao S., Fang X., Mu H., Yang H., Wang X., Xu Q., Gao H. (2015). Changes in fruit firmness, cell wall composition and cell wall degrading enzymes in postharvest blueberries during storage. Sci. Hortic..

[21] Silva M.-M., Silva E.-P., Garcia L.-G.-C., Asquieri E.-R., Boas E.-V.-B., Silva A.-P.-G., Xiao J., Damiani C. (2020). Bioactive compounds and nutritional value of cagaita (*eugenia dysenteric*) during its physiological development. eFood.

[22] Chen L., Zhou Y., He Z., Liu Q., Lai S., Yang H. (2018). Effect of exogenous ATP on the postharvest properties and pectin degradation of mung bean sprouts (*Vigna radiata*). Food Chem..

[23] Chang E.-H., Lee J.-S., Kim J.-G. (2017). Cell wall degrading enzymes activity is altered by high carbon dioxide treatment in postharvest ‘Mihong’ peach fruit. Sci. Hortic..

[24] Hou Y., Wu F., Zhao Y., Shi L., Zhu X. (2019). Cloning and expression analysis of polygalacturonase and pectin methylesterase genes during softening in apricot (*Prunus armeniaca* L.) fruit. Sci. Hortic..

[25] Dong Y., Zhang S., Wang Y. (2018). Compositional changes in cell wall polyuronides and enzyme activities associated with melting/mealy textural property during ripening following long-term storage of ‘Comice’ and ‘d’Anjou’ pears. Postharvest Biol. Technol..

[26] Bruno G.D., Troy E., María P., Orianne G., Reinaldo C.-V. (2018). Changes in cell wall pectins and their relation to postharvest mesocarp softening of "Hass" avocados (*Persea americana* Mill.). Plant Physiol. Bioch..

[27] Bunsiri A., Paull R.E., Ketsa S. (2012). Increased activities of phenyalanine ammonia lyase, peroxidase, and cinnamyl alcohol dehydrogenase in relation to pericarp hardening after physical impact in mangosteen (*Garcinia mangostana* L.). J. Hortic. Sci. Biotech..

[28] Ma Y., He H., Wu J., Wang C., Chao K., Huang Q. (2018). Assessment of polysaccharides from mycelia of genus ganoderma by mid-infrared and near-infrared spectroscopy. Sci. Rep..

[29] Humaira R., Najat A.B., Sarah A.A., Amal S.A., Rawan M.A., Horiah A.A. (2021). Postharvest disease management of Alternaria spots on tomato fruit by *Annona muricata* fruit extracts. Saudi. J. Biol. Sci..

[30] Wang M., Zhu P., Zhao S., Nie C., Wang N., Du X., Zhou Y. (2017). Characterization, antioxidant activity and immunomodulatory activity of polysaccharides from the swollen culms of *Zizania latifolia*. Int. J. Biol. Macromol..

[31] Wang Y., Wei X., Wang F., Xu J., Tang X., Li N. (2018). Structural characterization and antioxidant activity of polysaccharide from ginger. Int. J. Biol. Macromol..

[32] Zhang S., Hu T., Liu H., Chen Y., Pang X., Zheng L., Chang S., Kang Y. (2018). Moderate vacuum packing and low temperature effects on qualities of harvested mung bean (*Vigna radiata* L.) sprouts. Postharvest Biol. Technol..

[33] Wang D., Chen Q., Chen W., Guo Q., Xia Y., Wu D., Jing D., Liang G. (2021). Melatonin treatment maintains quality and delays lignification in loquat fruit during cold storage. Sci. Hortic..

[34] Gao Y., Chen H., Liu R., Wu W., Mu H., Han Y., Yang H., Gao H. (2022). Ameliorating effects of water bamboo shoot (*Zizania latifolia*) on acute alcoholism in a mice model and its chemical composition. Food Chem..

[35] Zhang Z., Li C., Zhang H., Ying Y., Hu Y., Song L. (2020). Comparative analysis of the lignification process of two bamboo shoots stored at room temperature. Plants.

[36] Chai T.-T., Xiao J., Sharmila M.-D., Wong F.-C. (2020). Laccase-catalyzed, phytochemical-mediated protein crosslinking conjugates. eFood.

[37] Sadat M., Kourosh M., Ali M., Fan W., Shu J. (2020). Increase in cell wall thickening and biomass production by overexpression of pmcesa2 in poplar. Front. Plant Sci..

[38] Ge Y., Zhang J., Li C., Xue W., Zhang S., Lv J. (2020). Trisodium phosphate delays softening of jujube fruit by inhibiting cell wall-degrading enzyme activities during ambient storage. Sci. Hortic..

[39] Ren Y., Sun P., Wang X., Zhu Z. (2020). Degradation of cell wall polysaccharides and change of related enzyme activities with fruit softening in *Annona squamosa* during storage. Postharvest Biol. Technol..

[40] Shi Z., Yang H., Jiao J., Wang F., Lu Y., Deng J. (2019). Effects of graft copolymer of chitosan and salicylic acid on reducing rot of postharvest fruit and retarding cell wall degradation in grapefruit during storage. Food Chem..

[41] Wang J., Jiang J., Wang J., Wang Z., Yang X., Jia L. (2019). The influence of gamma irradiation on the storage quality of bamboo shoots. Radiat. Phys. Chem..

[42] Ali S., Anjum M.A., Nawaz A., Naz S., Ejaz S., Sardar H., Saddiq B. (2020). Tragacanth gum coating modulates oxidative stress and maintains quality of harvested apricot fruits. Int. J. Biol. Macromol..

